# The origin of island populations of the African malaria mosquito, *Anopheles coluzzii*

**DOI:** 10.1038/s42003-021-02168-0

**Published:** 2021-05-26

**Authors:** Melina Campos, Mark Hanemaaijer, Hans Gripkey, Travis C. Collier, Yoosook Lee, Anthony J. Cornel, João Pinto, Diego Ayala, Herodes Rompão, Gregory C. Lanzaro

**Affiliations:** 1 Vector Genetics Laboratory, Department of Pathology, Microbiology and Immunology, UC Davis, Davis, CA USA; 2 Winclove Probiotics, Amsterdam, The Netherlands; 3 Florida Medical Entomology Laboratory, University of Florida, Vero Beach, FL USA; 4 Mosquito Control Research Laboratory, Department of Entomology and Nematology, University of California, Parlier, CA USA; 5 Global Health and Tropical Medicine, Instituto de Higiene e Medicina Tropical, Universidade Nova de Lisboa, Lisboa, Portugal; 6 MIVEGEC, IRD, CNRS, Université de Montpellier, Montpellier, France; 7 CIRMF, Franceville, Gabon; 8 Programa Nacional de Luta Contra o Paludismo, São Tomé, São Tomé and Príncipe

**Keywords:** Population genetics, Malaria

## Abstract

*Anopheles coluzzii* is a major malaria vector throughout its distribution in west-central Africa. Here we present a whole-genome study of 142 specimens from nine countries in continental Africa and three islands in the Gulf of Guinea. This sample set covers a large part of this species’ geographic range. Our population genomic analyses included a description of the structure of mainland populations, island populations, and connectivity between them. Three genetic clusters are identified among mainland populations and genetic distances (*F*_*ST*_) fits an isolation-by-distance model. Genomic analyses are applied to estimate the demographic history and ancestry for each island. Taken together with the unique biogeography and history of human occupation for each island, they present a coherent explanation underlying levels of genetic isolation between mainland and island populations. We discuss the relationship of our findings to the suitability of São Tomé and Príncipe islands as candidate sites for potential field trials of genetic-based malaria control strategies.

## Abstract (web summary)

Campos, Lanzaro and colleagues use whole-genome sequencing and population genomic analyses to infer connectivity between mainland and island mosquito populations in West Africa. The unique biogeographic history for each island population is reported, and the findings highlight potential candidate sites for genetic-based malaria control strategies.

## Introduction

From Darwin’s early work to the present, oceanic islands have served as model systems for the development of evolutionary theory. Attributes such as small size, distinct boundaries, and simplified biotas, together with relative youth and geographical isolation, have made islands a focus for the study of biological diversity^1^. Island remoteness is an obvious barrier for migration and one of the key factors in the theory of island biogeography, which relates island size and distance from the mainland to species richness^2^. Migration between related populations allows the exchange of heritable information and enhances genetic diversity, which is generally lower in island populations compared to mainland ones^3^.

From an applied perspective, geographically isolated sites are being considered for initial field trials of new genetic technologies applied to mosquito populations with the goal of malaria elimination. Isolation of a field site from non-target sites is generally considered a pivotal criterion in genetically engineered mosquitoes (GEM) field site selection. Emigration of GEMs out of the field trial site into neighboring, non-target sites on the mainland pose a problem especially as it relates to risk and regulatory concerns. Equally important is immigration of wild type individuals from neighboring sites into the trial site. Immigration in this case will confound efforts to measure GEM invasiveness and could potentially render the gene drive inefficient or even ineffective^4–6^. Malaria is a life-threatening parasitic disease, that in 2018 resulted in an estimated 405,000 deaths, of which 94% occurred in Africa^7^. Anopheline mosquitoes are responsible for transmitting malaria parasites to humans. In Africa, *Anopheles gambiae s.s* (hereafter *A. gambiae)* and *A. coluzzii* are among the principal vector species^8–10^. Malaria elimination strategies and interventions greatly rely on vector control methods^11^. However, modelling studies have shown that conventional vector control is insufficient for endemic malaria elimination^12,13^, which reinforces the conclusion that new methods, which may include GEM, are urgently needed^14–17^.

A thorough study of islands off the coast of Africa with the aim of identifying candidate sites for initial field trials of GEM has identified the country of São Tomé and Príncipe (STP) as a strong candidate^18^. This archipelago consists of two small oceanic islands in the Gulf of Guinea (West Africa), about 250 and 225 km, respectively, off the coast of Gabon. *Anopheles coluzzii* is thought to be the only malaria vector present on these islands^19,20^. Previous studies have shown genetic isolation between *A. coluzzii* populations from São Tomé and Príncipe islands, as well as between the island and mainland^19–22^ reinforcing the choice of STP as a suitable location for initial release of GEM. STP has recently reached the pre-elimination malaria level as defined by the World Health Organization^11^, due to the success of a combination of interventions, including indoor residual spraying, insecticide-treated nets, and artemisinin-based combination therapy^23–25^. Sustainability of these malaria vector control methods are challenged by limited financial support and decreased mosquito susceptibility to insecticides^25^.

Here we extend earlier studies describing genetic isolation between island and mainland *A. coluzzii* populations by applying analyses of 142 individual mosquito genomes. Using these data, we test the prediction that island populations are less genetically diverse than their mainland counterparts^3^, conduct an analysis of historical demography and assess ancestral patterns using cross-coalescence. We present genome resequencing data from three island populations: São Tomé, Príncipe and Bioko (Equatorial Guinea) and continental populations from nine African countries (Angola, Benin, Burkina Faso, Cotê d’Ivore, Gabon, Ghana, Guinea, Mali, and Cameroon). This sampling scheme covers the majority of *A. coluzzii*’s geographic distribution^26^. This is the first study using whole-genome sequencing to assess connectivity of conspecific populations on islands in the Gulf of Guinea with populations on the mainland and to explore the consequences of geography and geology on the genetics of these island populations. In addition, we consider our results as they relate to current and future vector control methods on the islands.

## Results

### Mosquito sampling and sequencing

The Vector Genetics Lab (VGL) dataset included 78 sequenced genomes. In total 2.4 billion reads were sequenced with a mean genome coverage of 12.6x per sample (Supplementary Tables 1 and 2). On average, 94.6% of reads were mapped to the reference genome. After joint variant calling and filtering for missingness, minimum depth and minimum allele frequency (MAF), we identified approximately 4.6 million accessible biallelic single-nucleotide polymorphisms (SNPs) across the whole genome. The dataset was expanded by the addition of 64 samples from the *A. gambiae* 1000 Genomes project phase 2 (The *Anopheles gambiae* 1000 Genomes Consortium—phase 2;^27^ Supplementary Table 2). After filtering for missingness and minor allele frequency, the combined dataset of 142 individuals contained 1,200,972 SNPs on the euchromatic regions of chromosome 3. Only biallelic SNPs from euchromatic regions on chromosome 3 were used to avoid confounding factors from common paracentric inversions on other chromosomes.

### Population structure

**Fig. 1:**
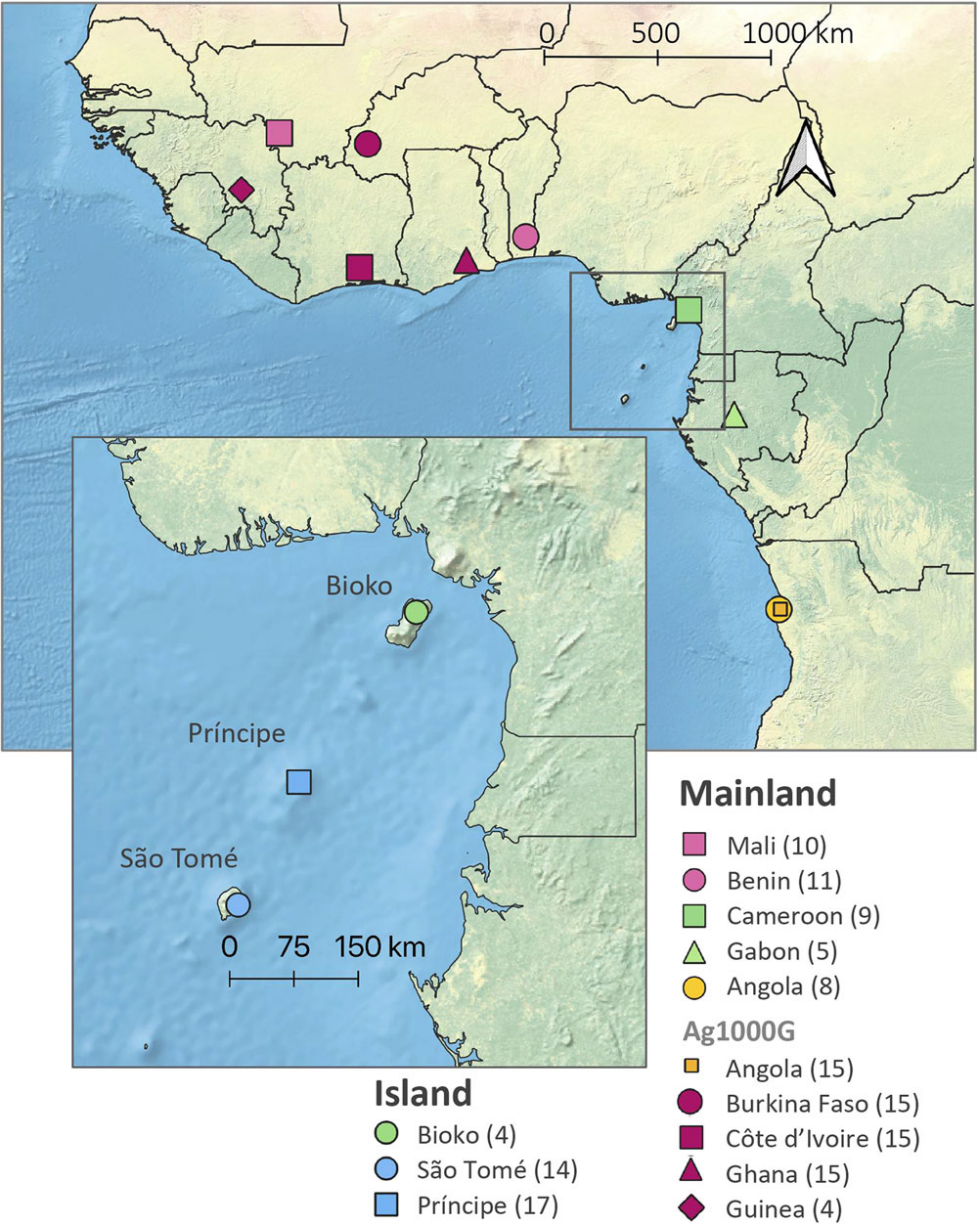
Sampling locations. Samples were collected by us from five countries in continental Africa: Angola (yellow dot), Benin (pink dot), Cameroon (green square), Gabon (green triangle), and Mali (pink square). The insert map of the Gulf of Guinea shows the three islands sampled: Bioko (green dot), São Tomé (blue dot), and Príncipe (blue square). Samples from four additional countries in continental Africa were included from the Ag1000G project (Miles et al., 2017): Burkina Faso (magenta dot), Cote d’Ivoire (magenta square), Ghana (magenta triangle), and Guinea (magenta rhombus); and additional samples from Angola (yellow square). The number of whole genome sequences analysed for each location is displayed in parenthesis. The CleanTOPO2 base map on QGIS was used as background.

**Fig. 2:**
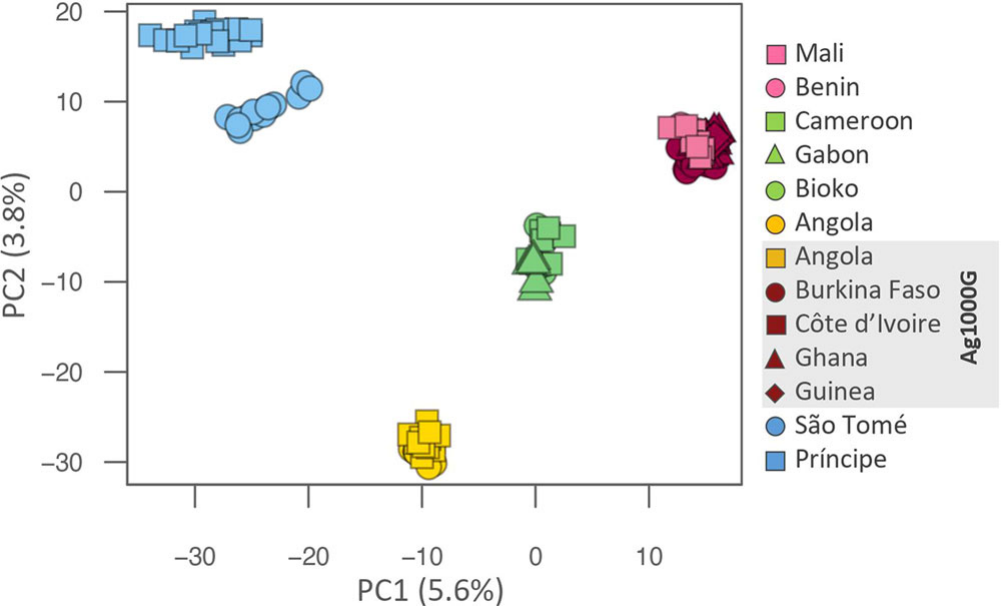
Principal component analysis. Plot of first two components of PCA. Analyses were based on 30,000 SNPs on chromosome 3 only. Island and mainland locations in West Africa are as in Fig. 1. Colours highlight the different clusters of mainland populations: Benin and Mali in pink plus Ag1000G populations in dark pink (Burkina Faso, Cote d’Ivoire, Ghana, and Guinea); Cameroon, Gabon, and Bioko in green; and Angola in yellow. São Tomé and Príncipe (STP) are separated from mainland populations by the first PC and between each other by PC2.

**Fig. 3:**
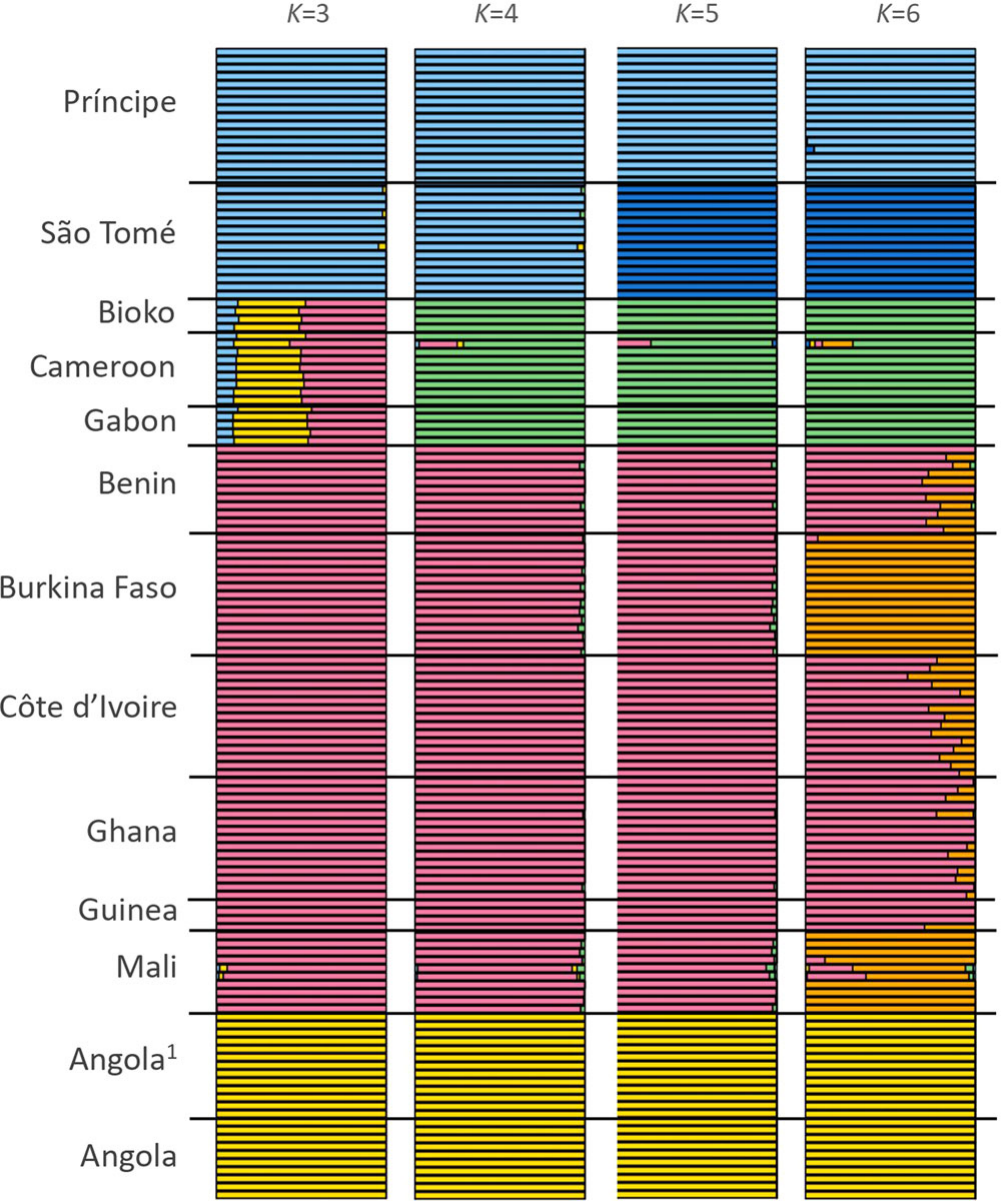
Bayesian analysis. Individual ancestry estimation with ADMIXTURE. Analyses were based on three independent replicates of 100,000 SNPs on chromosome 3 only. Samples were grouped by location. The lowest cross-validation error (CV error) value was for *K* = 3 (see Supplementary Fig. 1). *K* = 5 reveal similar relationships as those observed in the PCA results.

We investigated the genetic structure of *A. coluzzii* from island and mainland populations in West and Central Africa (Fig. 1). Both principal component and Bayesian clustering analyses suggest that populations on São Tomé and Príncipe islands are genetically differentiated from mainland populations (Figs. 2 and 3). Unlike STP, Bioko Island clusters with mainland populations from Gabon and Cameroon (Figs. 2 and 3). With *K* = 3 (lowest cross-validation error value for 1 < *K* < 10, Supplementary Fig. 1), STP samples belong to a genetic group distinct from all other populations included in this this study. When *K* is set to 4, a latitudinal clustering was observed among mainland populations i.e., a north-western group formed by Benin, Burkina Faso, Cotê d’Ivoire, Ghana, Guinea, and Mali, followed by Cameroon, Gabon and Bioko Island in central Africa, and a third cluster consisting of the samples from Angola (Fig. 3). Increasing *K* to 5 separates São Tomé and Príncipe populations. When *K* is set to 6 Mali plus Burkina Faso form a group distinct from the other north-western populations. Based on the results of these analysis, we recognize three mainland population groups: north-western, central (including Bioko), and southern. The same clustering pattern was derived using biallelic SNPs on the mitochondrial genome of these samples (Supplementary Figure 2, mitochondrial data was not available for Ag1000G populations).

**Fig. 4:**
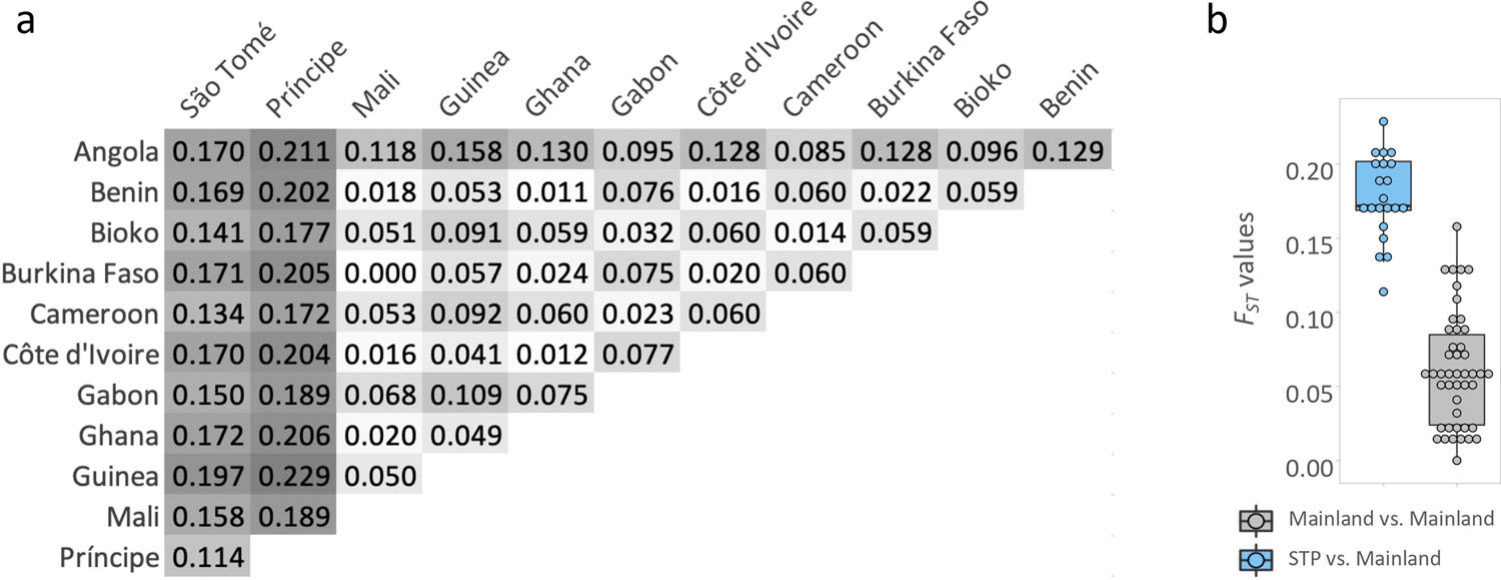
*F*_*ST*_ analyses. Pairwise *F*_*ST*_ between island and mainland locations in West Africa as in Fig. 1. **a** Heatmap table of pairwise *F*_*ST*_, higher values in darker grey. **b** Boxplot of test of *F*_*ST*_ median values of all mainland population comparisons (grey) and STP versus mainland populations comparisons (in blue).

**Fig. 5:**
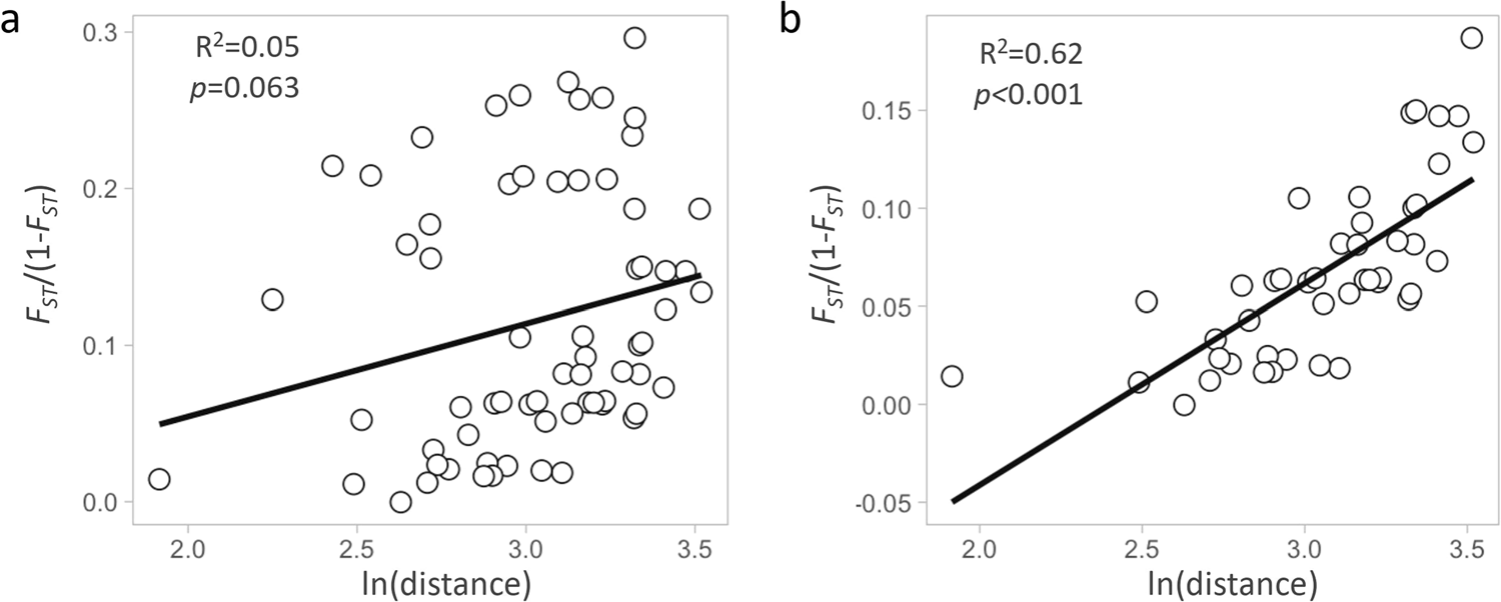
Isolation-by-distance test. Regression of genetic distance (*F*_*ST*_ /(1− *F*_*ST*_)) and logarithm of geographic distance. **a** All population pairwise comparisons. **b** Except São Tomé and Príncipe populations.

Mean *F*_*ST*_ between STP populations and mainland populations was significantly higher than *F*_*ST*_ among mainland populations only (Fig. 4a, b). Príncipe island was the most highly diverged (Fig. 4a). Regression tests for geographic distances and *F*_*ST*_ were uncorrelated if all population comparisons were included (*R*^2^ = 0.05, *p* = .063; Fig. 5) but significantly correlated when STP were excluded (*R*^2^ = 0.62, *p* < .001; Fig. 5).

### Population diversity

**Fig. 6:**
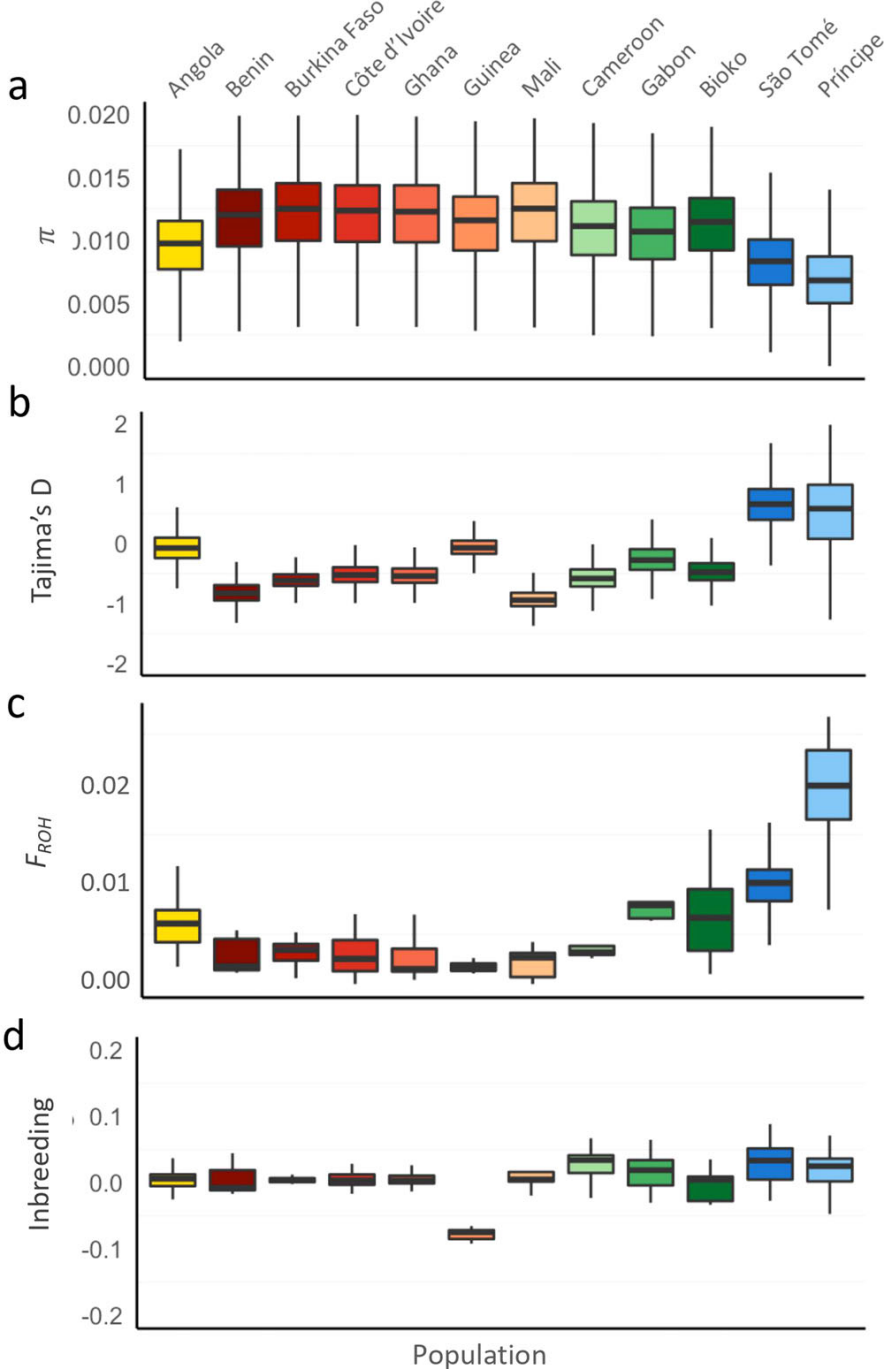
Population diversity. Metrics are grouped by sampling locations. **a** nucleotide diversity (π; in 20 kb windows) boxplot. **b** Tajima’s *D* (in 20 kb windows) boxplot. **c** Inbreeding statistic *F* (*F*_*IS*_) boxplot. **d** Length of runs of homozygosity (*F*_ROH_) boxplot. For all boxplots, the midline line is the median, with upper and lower limits (75^th^ and 25^th^ percentile, respectively), whiskers show maximum and minimum values and outliers are not shown.

Mean nucleotide diversity (π) was measured over the euchromatic regions of chromosome 3. Specimens from STP populations carried significantly less nucleotide diversity compared with mainland populations (*p* < 0.001; Fig. 6a): São Tomé median π was 0.83% and Príncipe 0.68%. Bioko island presented π similar to mainland populations (median π = 1.14%; *p* = 0.02). Tajima’s D statistic for *A. coluzzii* sequence from STP was *D* > 0, indicating a scarcity of rare alleles consistent with a population bottleneck which most likely occurred during the initial introduction of this species into the islands (founder event) and continued maintenance of small population size relative to populations on the mainland (Fig. 6b). All other populations presented Tajima’s *D* < 0. Populations on STP also showed significantly longer runs of homozygosity (*p* < 0.001; *F*_*ROH*_; Fig. 6c) and moderately higher inbreeding statistics (*p* = 0.002; *F*_*IS*_; Fig. 6d), whereas the population on Bioko island was similar to mainland populations for *F*_*ROH*_ (*p* = 0.18) and lower *F*_*IS*_ (*p* < 0.001). The results for STP populations are consistent with the hypothesis of reduced genetic diversity in remote oceanic island populations due to inbreeding and smaller population sizes.

### Population demographic history and cross-coalescence

**Fig. 7:**
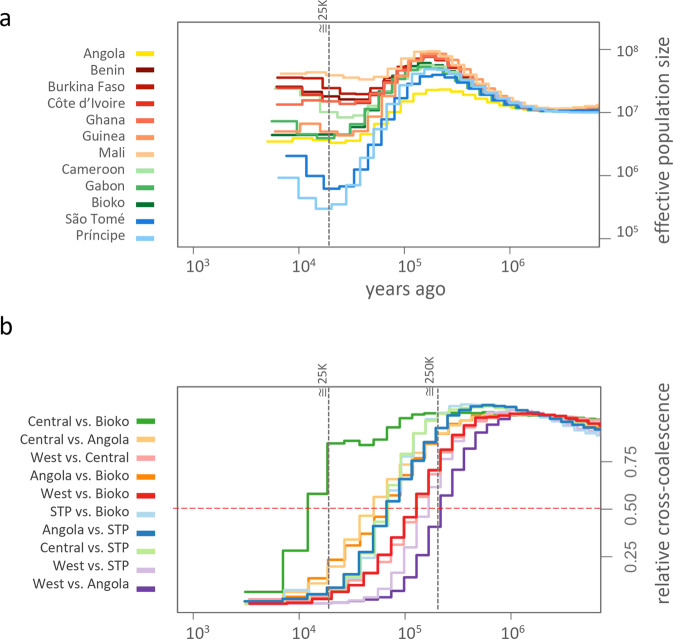
Effective population sizes and cross-coalescence estimates. **a** Historical effective population sizes for each population. The vertical line at about 25,000 years ago indicates the minimal turning point for the lowest population size. **b** Relative cross-coalescence (RCC) between island populations and the three genetic clusters found in the mainland: West (Burkina Faso, Benin, Côte d’Ivoire, Ghana, Guinea, and Mali), Central (Cameroon and Gabon), and Angola. The vertical grey line indicates the time point when effective population size was the lowest (top plot) and the first curve of RCC values reached below 0.5 values (red dashed line).

A reconstruction of the demographic history of *A. coluzzii* was created using Multiple sequentially Markovian coalescence (MSMC) analysis applied to our genome sequence data. The MSMC model uses large numbers (>100,000) of SNPs, each with its own coalescent i.e., time since the most recent common ancestor between the two alleles carried by an individual. The method reconstructs a demographic history from patterns in local density of heterozygous sites across the genome. As with other coalescence-based methods, MSMC can only infer scaled times using assumptions about numbers of generations/year (in our case we assume this is ten) and population sizes. These limitations are implicit where we refer to “years ago” and “effective population size (Ne)”. Prior to about 900,000 years ago the effective population size of a putative ancestral population was relatively large (N_e_~10^7^) and stable (Fig. 7a). From that point forward populations or population groups began following distinct demographic trajectories. Five of six western populations (Guinea is the exception) experienced initial size expansion subsequentially stabilizing and remaining relatively large, in which Mali is the largest. Central African populations (Bioko, Cameroon, and Gabon), and Guinea also experienced size expansion. With the exception of Cameroon, they subsequently followed a declining trajectory stabilizing at an intermediate level. The southern (Angola) population settled at an intermediate Ne about 50,000 years ago but experienced the smallest size expansion among the continental populations. Populations of *A. coluzzii* from São Tomé and Príncipe experienced a dramatic population bottleneck (founder effect), which likely occurred during the process of colonizing the islands. Approximately 25,000 years ago, the STP populations reached their nadir, followed by steady increase, however, their Ne remained lower than any other population in this study.

Shared population history was estimated using cross-coalescence. A higher relative cross-coalescence (RCC) indicates less time to the last common ancestor shared by the two populations in a specific pair-wise comparison (Fig. 7b). Heuristically, the time that two populations diverged is ascertained when RCC equals 0.5 (the mid-point of that decline). All populations shared common ancestors in the deep past, reflecting high connectivity as one great population. About ~200,000 years ago, RCC between the west African group and all other populations started decreasing considerably, reaching the mid-point or below first for Angola, then STP, followed by Bioko and the central African cluster (Fig. 7b). RCC between STP and mainland populations decline over time, and the islands became fully isolated (RCC = 0) about ~25,000 years ago, this corresponds with the point they reached the lowest population size (Fig. 7a). At the same timepoint, Bioko presents RCC as high as ~0.8 with central African populations (Fig. 7b), indicating considerable gene flow among them.

## Discussion

Dispersal of malaria vector species has been extensively explored because it directly affects disease transmission, the spread of insecticide resistance and the development of control strategies^28,29^. Mosquito dispersal can be measured by conventional mark–release-recapture experiments for short range movement^30,31^, directly by air-borne insect sampling for long distances^29^ or through estimation of gene flow between populations applied at various scales. Here we describe important aspects of *A. coluzzii* dispersal and historical phylogeography using a population genomics approach. This is the first whole-genome resequencing study covering a large part of this species’ range focusing on island as well as mainland populations.

*Anopheles coluzzii* samples from mainland populations were consistently divided into three geographically related population groups: (i) Benin, Burkina Faso, Cotê d’Ivoire, Ghana, Guinea, and Mali forming a western group, (ii) Cameroon and Gabon forming a central group and (iii) Angola representing a southern group (Figs. 2 and 3).

*Anopheles coluzzii* overlaps with its sister species *A. gambiae* over 90% of its geographical range, which includes Central and West Africa^26^. The two species are very closely related genetically and hybrids between the two in nature have been frequently reported^32–36^. Despite their similarities, the relationship among intraspecific populations of the two species appear substantially different. *A. gambiae* has a shallow population structure over its broad distribution spanning sub-Saharan Africa^37–39^. Our analysis of populations spanning the distribution of *A. coluzzii* revealed positive isolation by distance (Fig. 5b), corroborating previous reports^27,40^. The two species differ with respect to the types of aquatic habitats occupied by the larval stages. Whereas *A. coluzzii* larvae inhabit semi-permanent bodies of water, generally associated with agriculture *A. gambiae* is more typically found in temporary rain-dependent water bodies^41,42^. In the Sahel, where there is a pronounced dry season, recent studies have suggested that *A. coluzzii* persists through dry seasons via dormancy (aestivation), whereas *A. gambiae* populations experience local extinctions followed by reestablishment via long-distance migration^43–45^. These observations suggest that *A. gambiae* has a greater capacity for dispersal compared with its sister species *A. coluzzii*.

Based on our analyses of historical population size and cross-coalescence, we hypothesize that, like *A. gambiae*^46^ the geographical origin of *A. coluzzii* was from a west African ancestral population represented by populations in Mali (largest in size), Burkina Faso, Benin, Cotê d’Ivoire, and Ghana (Fig. 7a). The west African and Cameroonian populations have no sign of strong historical fluctuations in population size, whereas populations in Guinea, Gabon, and Angola experienced a decrease in effective population size. Concerning the cross-coalescence analysis, we observed a split that occurred ~200,000 years ago separating west African populations (Mali and Benin) first from Angola, then from the others (Fig. 7b), consistent with vicariance in *A. gambiae* populations where the Congo River basin acts as a geological barrier to dispersal^46,47^.

The genomes of *A. coluzzii* from São Tomé and Príncipe islands bear signatures consistent with the biogeography of remote oceanic island populations i.e., reduced genetic diversity, signs of inbreeding and low population size. The genomes of Bioko island population were similar to those on the mainland (Figs. 6 and 7a). All three are located in the Gulf of Guinea as part of the chain of volcanoes of the Cameroonian line^48^. However, Bioko is only 32 km off the coast of Gabon whilst São Tomé and Príncipe are 250 and 225 km from Gabon respectively. Beyond simply distance from the African mainland, important biogeographic aspects differentiate Bioko from São Tomé and Príncipe islands and these are reflected by the biology of the organisms inhabiting these islands.

Bioko is a land-bridge island, lying on the continental shelf in shallow seas only 60 m deep^49,50^. Sea levels were historically lowered sufficiently to connect Bioko to the Africa mainland during the last glaciation^49^. In contrast, São Tomé and Príncipe are oceanic islands that have never been connected with the mainland nor with each other, and are separated by seas over 1800 m deep^49^. Reflecting its continental origin, Bioko’s fauna and flora are relatively species-rich, but with low levels of species endemism, explained by its former connection to the mainland and short period of isolation^49–51^. São Tomé and Príncipe islands present an inverted pattern i.e. high endemism, that includes the mosquito fauna^49,52^, and low species richness. In STP only two species of anopheline mosquitoes have been reported, *A. coluzzii* and *A. coustani*^19,53^, whilst on Bioko there are at least five: *A. gambiae*, *A. coluzzii*, *A. melas*, *A. funestus*, and *A. smithii*^54,55^.

Regarding connectivity between island and mainland populations, we found strong evidence that *A. coluzzii* from STP islands are isolated from mainland populations, while samples from Bioko are closely related to central African populations from Cameroon and Gabon. These results were supported by population structure analysis using PCA (Fig. 2), admixture (Fig. 3), and pairwise *F*_*ST*_ (Fig. 4a). Considerably higher genetic divergence was found between São Tomé island or Príncipe island populations and those on the mainland than among mainland populations (*p* < 0.001; Fig. 4b). In addition, divergence was high between the two islands (*F*_*ST*_ = 0.11); and admixture analysis (*K* = 5) assigned each island to a distinct genetic cluster (Fig. 3).

The results we report here are consistent with and extend earlier work on the genetic structure of mainland and island populations of *A. coluzzii* around the Gulf of Guinea. This earlier work described the genetic structure of populations using microsatellite markers^19,20^, mitochondrial ND5, rDNA internal transcribed spacer sequences^21^ and transposable element (*Herves*) insertion site polymorphisms^22^. Collectively these works revealed genetic isolation between populations in STP and Gabon and little isolation between populations on Bioko and the mainland.

Our analysis shows clear isolation of STP *A. coluzzii* populations from those on the African mainland and suggests that these populations were diverged about ~25,000 years ago (RCC = 0). Analysis of populations on Bioko indicate recent (RCC~0.8; Fig. 7b) and perhaps contemporary gene flow with those in central African. This observation agrees with the geological history of Bioko which became isolated from the mainland by rising sea levels only ~11,000 years ago^49^. São Tomé and Príncipe island populations experienced a sharp decrease in size, suggesting that a small portion of the ancestral population became established there (founder effect), whereas the population size trajectory on Bioko is similar to the mainland (Fig. 7a). Of note, exact time in years could change if the assumptions for mutation rate and number of generations per year are revised, however relative separation remain relevant.

In this study, we used a population genomics analysis to explore the relationship between malaria mosquito populations on the remote oceanic islands of São Tomé and Príncipe with mainland populations bordering the Gulf of Guinea in west Africa. Our results are consistent with studies of mosquitoes on other oceanic islands^27,46^. Similar work analyzing anopheline mosquitoes on lacustrine islands in Lake Victoria suggest a much lower degree of genetic isolation^56^, which is not surprising considering their geographic proximity to the mainland.

Population genetic studies are vital for improving the design and organization of vector control strategies, including and especially field trials of genetically engineered mosquitoes. Genetic control methods offer potential for low-cost, sustainable malaria elimination in highly endemic areas where conventional methods have shown to be insufficient^12,13^. In order to best evaluate the performance of modified mosquitoes, a confined field trial site is required, defined by minimal gene flow between neighboring populations. Here we show that populations of *A. coluzzii* from the islands of São Tomé and Príncipe are genetically isolated, both from each other and from the nearest mainland populations. Previous studies have reported isolation of these islands using fewer genetic markers^19,21,22^. We have expanded this work by analyzing individual mosquito whole genome sequences from a wide range of *A. coluzzii* populations on the mainland for comparison. Our population genomics approach also allowed us to explore the evolution of island populations in terms of ancestry and demography. Future work on populations of malaria vectors in São Tomé and Príncipe will focus on the relationship among *A. coluzzii* sub-populations within each island.

## Methods

### Population sampling

In this paper we describe a population genomics analysis of two sets of genome sequence data. One dataset includes data generated from samples collected from field sites by the Vector Genetics Laboratory (VGL) at UC Davis. These samples included adult and larval stage *A. coluzzii* specimens (*N* = 78) collected from field sites using standard methods (Marsden et al. 2011, Moreno 2003) and archived at the VGL, UC Davis, and at the Instituto de Higiene e Medicina Tropical (IHMT), Universidade Nova de Lisboa, Portugal (Fig. 1; Supplementary Table 1). This set of specimens included samples from eight localities: three islands in the Gulf of Guinea (*N* = 4 from Bioko, *N* = 14 from São Tomé and *N* = 17 from Príncipe), four Gulf of Guinea coastal mainland sites (*N* = 8 from Angola, *N* = 11 from Benin, *N* = 9 from Cameroon, and *N* = 5 from Gabon) and one inland site (Mali *N* = 10). Species diagnostics was performed using species-specific markers included in the divergence island SNPs (DIS) assay as described in Lee et al.^57^. In addition to these 78 samples, we included genome sequence data from 64 individuals taken from the publicly available Ag1000 database (The *Anopheles gambiae* 1000 Genomes Consortium—phase2^27^). These included samples of *A. coluzzii* from five countries: Angola, Burkina Faso, Cotê d’Ivoire, Ghana, and Guinea. Analysis was performed with 15 samples from each country except for *N* = 4 from Guinea (Supplementary Table 2).

### Whole genome sequencing

Individual mosquito DNA from the VGL samples was extracted using a Qiagen Biosprint following our established protocol^58^. DNA yield was measured using a dsDNA high sensitivity assay kit on a Qubit instrument (Thermo Fisher Scientific, Waltham, MA, USA). The KAPA HyperPlus Kit (Roche Sequencing Solutions, Indianapolis, Indiana, USA) was used for individual genomic DNA libraries using 10 ng DNA as input, as described in Yamasaki, et al.^59^. Size selection of the libraries and clean-up was performed using AMPure SPRI beads (Beckman Coulter Life Sciences, Indianapolis, Indiana, USA). Individual library concentrations were measured using Qubit and then pooled in equal amounts for sequencing using an Illumina HiSeq 4000 instrument at the UC Davis DNA Technologies Core facility. Methods used for genome sequencing of individuals from the Ag1000 samples are described elsewhere (Clarkson et al., 2020).

### Data processing, mapping, and variant calling

After filtering and trimming demultiplexed raw reads using Trimmomatic v0.36^60^, the VGL sample reads were mapped to the reference AgamP4^61,62^ using BWA-MEM v0.7.15^63^ with default settings. Duplicate reads were removed using Sambamba markdup^64^. Freebayes v1.2.0^65^ was used for variant calling, with standard filters and the “-no-population-priors”, “theta = 0.01”, and “max-comple-gap = 0” options. Variants were normalized with *vt normalize* v0.5^66^ and those without support from both overlapping forward and reverse reads were removed using vcffilter v1.0.0rc2 (https://github.com/vcflib/vcflib). Only biallelic SNPs with minimum depth of 8 and maximum of 5% of missing data were used for further analysis.

### Mitochondria

Mitogenome variant calling were generated assuming single ploidy using Freebayes v1.2.0. Singletons and SNPs in an AT-rich region were removed from further analysis due to low coverage^67^. A neighbor-joining tree was constructed with Nei’s distance matrix and 1000 bootstrap replicates using the R package *ape* 5.4^68^.

### Population structure

The VGL dataset was merged with biallelic SNP data from Ag1000G using BCFtools v1.9^69^ followed by restrictive filtering. We removed any SNP that did not pass the accessibility filter of the Ag1000G dataset, any SNPs with >10% missingness, and SNPs with minor allele frequency (MAF) < 1%. Also, population structure analysis was based on chromosome 3 SNPs only. This was done to avoid confounding signals from polymorphic inversions on chromosomes 2 and X^62^. Heterochromatic regions on chromosome 3 R (3 R:38,988,757-41,860,198; 3 R:52,161,877-53,200,684) and 3 L (3 L:1-1,815,119; 3 L:4,264,713-5,031,692) were also filtered out^62^.

Principal component analysis (PCA) was performed after pruning for LD using scikit-allel v1.2.0^70^. Hudson’s estimator^71,72^ was used for pairwise fixation indices *F*_*ST*_ calculation implemented in scikit-allel v1.2.0. Isolation-by-distance was tested using the regression of *F*_*ST*_/(1−*F*_*ST*_) estimates against the logarithm of geographical distance^73^ in R. Population structure was also explored by assignment of individual genomes to ancestry components using ADMIXTURE v1.3.0^74^. A total of three independent replicate samples of 100,000 SNPs (<10% of the full dataset) from chromosome 3 were submitted for admixture analysis. For each replicate, ten iterations were performed for values of *K* clusters from 1 to 10, resulting in 30 estimates per *K*. The results were compiled using the online version of CLUMPAK and plotted in R. Best-fitting *K* was determined by the lowest cross-validation error values.

Nucleotide diversity (π) and Tajima’s D were calculated in nonoverlapping windows of 20 kb on euchromatic regions of chromosome 3 using VCFtools^75^. VCFtools was also used for the calculation of inbreeding statistics (*F*_*IS*_) using the method-of-moments approach. Runs of homozygosity (ROH) within each individual were inferred outside inversions and heterochromatic regions and LD-pruned SNPs on chromosome 3 set using PLINK v1.9^76^, requiring 10 homozygous SNPs spanning a distance of 100 kb and default parameters. The results were grouped by population and significance tests performed between the islands of São Tomé and Príncipe (STP) and the remaining populations under study or Bioko and mainland categories using a Wilcoxon rank-sum test in R.

### Population sizes and cross-coalescence analysis

Population size estimation and cross-coalescence analyses were performed using the multiple sequentially Markovian coalescent (MSMC) pipeline MSMC2 v2.0.2^77^ following the author’s protocol (https://github.com/stschiff/msmc2). For this analysis, SNPs on chromosome 3 R and 3 L were phased with SHAPEIT2 v2.9^78^ using an *A. gambiae* recombination map^39^. Heterochromatic regions on chromosome 3 R and 3 L were removed after phasing. Four samples per population were randomly selected and used for population size and two per population or genetic cluster for cross-coalescence inter-population with 20 Baum–Welch iterations each. The results were plotted in R, converting the results to real time in years and assuming 10 generations per year and mutation rate of 2.85 × 10^−9^ (median between mutation rates in insects as used in Schmidt, et al.^46^).

### Statistics and reproducibility

One hundred and forty-two specimens of *A. coluzzii* from 12 populations from Africa were used for this study. Statistical analyses were performed in R and corresponding *p* values are reported in the text and/or figures.

### Reporting summary

Further information on research design is available in the Nature Research Reporting Summary linked to this article.

## Supplementary information

Supplementary Information

Reporting Summary

## Data Availability

New whole genome sequence data included in this study are deposited in NCBI GenBank with accession numbers SAMN17251765, SAMN15641374- SAMN15641426 under BioProject ID PRJNA648422.
