# Supplementary material for: The origin of island populations of the African malaria mosquito, Anopheles coluzzii
Source: Commun Biol. 2021 May 26;4:630. doi: 10.1038/s42003-021-02168-0 (PMC8155153; doi:10.1038/s42003-021-02168-0)
Supplement: Supplementary file 1 — Supplementary Information [file 42003_2021_2168_MOESM1_ESM.pdf]

Supplementary Information

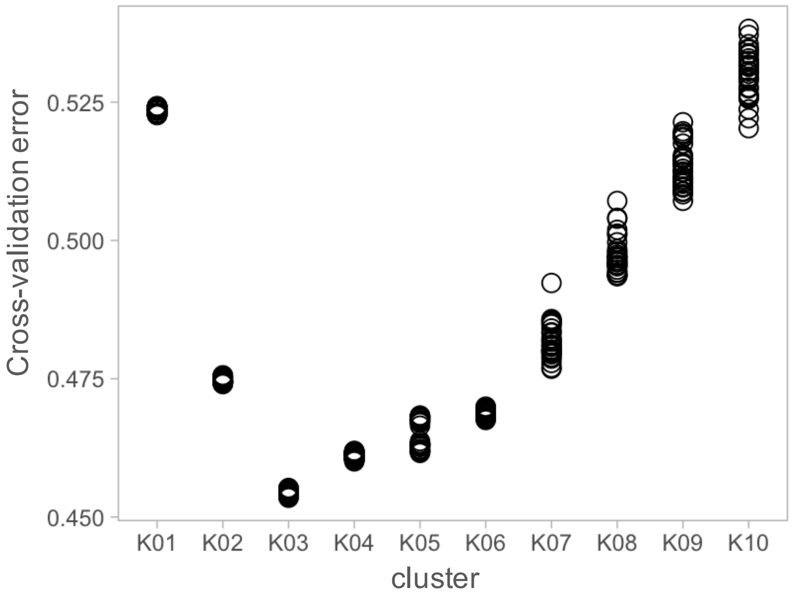

Supplementary Figure 1. Cross-validation error. Values of cross-validation error for *K* from 1 to 10 in ADMIXTURE analysis.

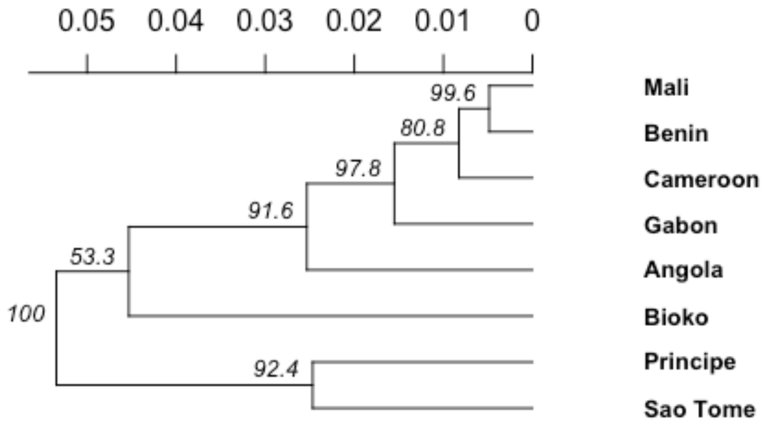

Supplementary Figure 2. Neighbour-joining tree. Neighbour-joining tree was constructed using mitogenome variants of *Anopheles coluzzii* populations. Number on each branch is the percentage of times among 1,000 replicates.

Supplementary Table 1. Metadata per sample.

Sample-ID	Locality	Country	Population	Category	Year	Latitude	Longitude
6617_P	Príncipe	São Tomé and Príncipe	Príncipe	island	2017	1.63	7.42
6917_K	Príncipe	São Tomé and Príncipe	Príncipe	island	2017	1.63	7.42
0117_A	Alto Doro	São Tomé and Príncipe	São Tomé	island	2017	0.20	6.71
0117_J	Alto Doro	São Tomé and Príncipe	São Tomé	island	2017	0.20	6.71
0117_N	Alto Doro	São Tomé and Príncipe	São Tomé	island	2017	0.20	6.71
10217_H	Alto Doro	São Tomé and Príncipe	São Tomé	island	2017	0.20	6.71
10217_I	Alto Doro	São Tomé and Príncipe	São Tomé	island	2017	0.20	6.71
10617_L	Alto Doro	São Tomé and Príncipe	São Tomé	island	2017	0.20	6.71
10617_N	Alto Doro	São Tomé and Príncipe	São Tomé	island	2017	0.20	6.71
0117_D	Alto Doro	São Tomé and Príncipe	São Tomé	island	2017	0.20	6.71
10217_L	Alto Doro	São Tomé and Príncipe	São Tomé	island	2017	0.20	6.71
6217_G	Príncipe	São Tomé and Príncipe	Príncipe	island	2017	1.63	7.42
6317_R	Príncipe	São Tomé and Príncipe	Príncipe	island	2017	1.63	7.42
6317_W	Príncipe	São Tomé and Príncipe	Príncipe	island	2017	1.63	7.42
6417_K	Príncipe	São Tomé and Príncipe	Príncipe	island	2017	1.63	7.42
6617_B	Príncipe	São Tomé and Príncipe	Príncipe	island	2017	1.63	7.42
8017_D	Príncipe	São Tomé and Príncipe	Príncipe	island	2017	1.63	7.42
6017_C	Príncipe	São Tomé and Príncipe	Príncipe	island	2017	1.63	7.42
6317_Z	Príncipe	São Tomé and Príncipe	Príncipe	island	2017	1.63	7.42
02BIOK020	Bioko	Equatorial Guinea	Bioko	mainland	2002	3.61	8.78
02BIOK021	Bioko	Equatorial Guinea	Bioko	mainland	2002	3.61	8.78
02BIOK024	Bioko	Equatorial Guinea	Bioko	mainland	2002	3.61	8.78
02BIOK028	Bioko	Equatorial Guinea	Bioko	mainland	2002	3.61	8.78
03TIKO0027	Tiko	Cameroon	Cameroon	mainland	2003	4.07	9.36
03TIKO0032	Tiko	Cameroon	Cameroon	mainland	2003	4.07	9.36
03TIKO0052	Tiko	Cameroon	Cameroon	mainland	2003	4.07	9.36
03TIKO0059	Tiko	Cameroon	Cameroon	mainland	2003	4.07	9.36
11TIKO128	Tiko	Cameroon	Cameroon	mainland	2011	4.07	9.36
11TIKO326	Tiko	Cameroon	Cameroon	mainland	2011	4.07	9.36
11TIKO335	Tiko	Cameroon	Cameroon	mainland	2011	4.07	9.36
11TIKO391	Tiko	Cameroon	Cameroon	mainland	2011	4.07	9.36
11TIKO403	Tiko	Cameroon	Cameroon	mainland	2011	4.07	9.36
98PRIN003	Príncipe	São Tomé and Príncipe	Príncipe	island	1998	1.63	7.42
98PRIN006	Príncipe	São Tomé and Príncipe	Príncipe	island	1998	1.63	7.42
98PRIN010	Príncipe	São Tomé and Príncipe	Príncipe	island	1998	1.63	7.42
98PRIN011	Príncipe	São Tomé and Príncipe	Príncipe	island	1998	1.63	7.42
98PRIN026	Príncipe	São Tomé and Príncipe	Príncipe	island	1998	1.63	7.42
98PRIN044	Príncipe	São Tomé and Príncipe	Príncipe	island	1998	1.63	7.42
98PRIN046	Príncipe	São Tomé and Príncipe	Príncipe	island	1998	1.63	7.42
98SAOT002	São Tomé	São Tomé and Príncipe	São Tomé	island	1998	0.20	6.71

98SAOT016	São Tomé	São Tomé and Príncipe	São Tomé	island	1998	0.20	6.71
98SAOT018	São Tomé	São Tomé and Príncipe	São Tomé	island	1998	0.20	6.71
98SAOT027	São Tomé	São Tomé and Príncipe	São Tomé	island	1998	0.20	6.71
98SAOT041	São Tomé	São Tomé and Príncipe	São Tomé	island	1998	0.20	6.71
2018GABO008	Gabon	Gabon	Gabon	mainland	2018	-0.43	11.33
2018GABO009	Gabon	Gabon	Gabon	mainland	2018	-0.43	11.33
2018GABO010	Gabon	Gabon	Gabon	mainland	2018	-0.43	11.33
2018GABO011	Gabon	Gabon	Gabon	mainland	2018	-0.43	11.33
2018GABO013	Gabon	Gabon	Gabon	mainland	2018	-0.43	11.33
2018_LU52_14	Luanda	Angola	Angola	mainland	2010	-8.83	13.26
2018_LU54_14	Luanda	Angola	Angola	mainland	2010	-8.83	13.26
2018_LU55_14	Luanda	Angola	Angola	mainland	2010	-8.83	13.26
2018_LU56_14	Luanda	Angola	Angola	mainland	2010	-8.83	13.26
2018_LU57_14	Luanda	Angola	Angola	mainland	2010	-8.83	13.26
2018_LU58_14	Luanda	Angola	Angola	mainland	2010	-8.83	13.26
2018_LU59_14	Luanda	Angola	Angola	mainland	2010	-8.83	13.26
2018_LU60_14	Luanda	Angola	Angola	mainland	2010	-8.83	13.26
2014TOR002	Tori	Benin	Tori	mainland	2014	6.56	2.24
2014TOR004	Tori	Benin	Tori	mainland	2014	6.56	2.24
2014TOR007	Tori	Benin	Tori	mainland	2014	6.56	2.24
2014TOR008	Tori	Benin	Tori	mainland	2014	6.56	2.24
2014TOR010	Tori	Benin	Tori	mainland	2014	6.56	2.24
2014TOR011	Tori	Benin	Tori	mainland	2014	6.56	2.24
2014ABO003	Abomey	Benin	Cove	mainland	2014	7.22	2.34
2014COV001	Cove	Benin	Cove	mainland	2014	7.22	2.34
2014COV002	Cove	Benin	Cove	mainland	2014	7.22	2.34
2014COV004	Cove	Benin	Cove	mainland	2014	7.22	2.34
2014COV006	Cove	Benin	Cove	mainland	2014	7.22	2.34
02SELI0080	Selinkenyi	Mali	Mali	mainland	2002	11.70	-8.28
02SELI0084	Selinkenyi	Mali	Mali	mainland	2002	11.70	-8.28
02SELI0085	Selinkenyi	Mali	Mali	mainland	2002	11.70	-8.28
04SELI0084	Selinkenyi	Mali	Mali	mainland	2004	11.70	-8.28
06SELI0015	Selinkenyi	Mali	Mali	mainland	2006	11.70	-8.28
06SELI0030	Selinkenyi	Mali	Mali	mainland	2006	11.70	-8.28
10SELI_O124	Selinkenyi	Mali	Mali	mainland	2010	11.70	-8.28
10SELI_O160	Selinkenyi	Mali	Mali	mainland	2010	11.70	-8.28
12SELI0021	Selinkenyi	Mali	Mali	mainland	2012	11.70	-8.28
12SELI0072	Selinkenyi	Mali	Mali	mainland	2012	11.70	-8.28

Supplementary Table 2. Statistic and accession number per sample.

Sample-ID	Raw reads	Mapped reads	Mapping%	Median insert	Deapth(x)	Accession	Bioproject
6617_P	27607034	26896795	97.43%	380	11.6	SAMN15641374	PRJNA648422
6917_K	19927555	19727561	99.00%	427	8.8	SAMN15641375	PRJNA648422
0117_A	23220548	22717672	97.83%	411	9.8	SAMN15641376	PRJNA648422
0117_J	36074661	35565508	98.59%	435	15.2	SAMN15641377	PRJNA648422
0117_N	39381103	37492322	95.20%	426	16.1	SAMN15641378	PRJNA648422
10217_H	22181368	19815950	89.34%	379	8.6	SAMN15641379	PRJNA648422
10217_I	29999128	26839914	89.47%	405	11.9	SAMN15641380	PRJNA648422
10617_L	20375421	19433567	95.38%	413	8.6	SAMN15641381	PRJNA648422
10617_N	23493748	22696708	96.61%	401	10.0	SAMN15641382	PRJNA648422
0117_D	25428933	24060279	94.62%	418	10.5	SAMN15641383	PRJNA648422
10217_L	40370969	37491915	92.87%	415	15.8	SAMN15641384	PRJNA648422
6217_G	21347377	20726245	97.09%	385	9.2	SAMN15641385	PRJNA648422
6317_R	40074137	39480624	98.52%	405	16.3	SAMN15641386	PRJNA648422
6317_W	22568166	22112727	97.98%	417	9.5	SAMN15641387	PRJNA648422
6417_K	49899512	48715081	97.63%	362	20.9	SAMN15641388	PRJNA648422
6617_B	23902490	23640625	98.90%	424	10.3	SAMN15641389	PRJNA648422
8017_D	33507344	32190466	96.07%	381	13.7	SAMN15641390	PRJNA648422
6017_C	26311925	26046914	98.99%	383	11.5	SAMN15641391	PRJNA648422
6317_Z	23672855	22896916	96.72%	393	10.2	SAMN15641392	PRJNA648422
02BIOK020	25058247	24683129	98.50%	340	11.1	SAMN14119535	PRJNA607000
02BIOK021	23343962	20693091	88.64%	327	9.1	SAMN14119536	PRJNA607000
02BIOK024	23983649	23648758	98.60%	353	10.9	SAMN17251765	PRJNA648422
02BIOK028	31291988	30958559	98.93%	349	13.5	SAMN14119537	PRJNA607000
03TIKO0027	47293003	29252269	61.85%	400	11.0	SAMN15641393	PRJNA648422
03TIKO0032	26612400	22325703	83.89%	438	8.6	SAMN15641394	PRJNA648422
03TIKO0052	35336653	23779234	67.29%	425	9.1	SAMN15641395	PRJNA648422
03TIKO0059	31202501	24373175	78.11%	438	9.2	SAMN15641396	PRJNA648422
11TIKO128	50009375	48428438	96.84%	386	20.3	SAMN15641401	PRJNA648422
11TIKO326	29671688	28277814	95.30%	412	10.8	SAMN15641400	PRJNA648422
11TIKO335	24595173	24232761	98.53%	430	9.0	SAMN15641399	PRJNA648422
11TIKO391	28461831	28099817	98.73%	401	10.4	SAMN15641398	PRJNA648422
11TIKO403	29862635	23562961	78.90%	379	9.1	SAMN15641397	PRJNA648422
98PRIN003	51684908	51104328	98.88%	399	20.7	SAMN15641402	PRJNA648422
98PRIN006	25836339	25532534	98.82%	403	9.5	SAMN15641403	PRJNA648422
98PRIN010	27243521	26930864	98.85%	414	10.2	SAMN15641404	PRJNA648422
98PRIN011	23595113	23349506	98.96%	398	8.7	SAMN15641405	PRJNA648422
98PRIN026	25110274	24858111	99.00%	418	9.4	SAMN15641406	PRJNA648422
98PRIN044	24133905	23862450	98.88%	408	9.1	SAMN15641407	PRJNA648422
98PRIN046	27408087	25943082	94.65%	399	10.4	SAMN15641408	PRJNA648422
98SAOT002	26619380	24679185	92.71%	430	8.9	SAMN15641409	PRJNA648422

98SAOT016	49359395	48802357	98.87%	412	19.3	SAMN15641410	PRJNA648422
98SAOT018	33987213	33584711	98.82%	411	12.0	SAMN15641411	PRJNA648422
98SAOT027	25716350	25455425	98.99%	427	9.4	SAMN15641412	PRJNA648422
98SAOT041	32081658	31195602	97.24%	408	11.7	SAMN15641413	PRJNA648422
2018GABO008	50348481	49730105	98.77%	390	20.6	SAMN15641415	PRJNA648422
2018GABO009	28242139	27943444	98.94%	397	10.9	SAMN15641414	PRJNA648422
2018GABO010	28324062	28009018	98.89%	396	10.1	SAMN15641416	PRJNA648422
2018GABO011	29017908	28256451	97.38%	380	10.0	SAMN15641417	PRJNA648422
2018GABO013	27255034	26987890	99.02%	411	10.0	SAMN15641418	PRJNA648422
2018_LU52_14	31977718	31512146	98.54%	350	14.6	SAMN15641421	PRJNA648422
2018_LU54_14	32694703	31580956	96.59%	341	14.5	SAMN15641422	PRJNA648422
2018_LU55_14	34296708	33891236	98.82%	331	15.8	SAMN15641423	PRJNA648422
2018_LU56_14	34385798	33681803	97.95%	358	15.8	SAMN15641419	PRJNA648422
2018_LU57_14	38143026	37242756	97.64%	334	17.0	SAMN15641424	PRJNA648422
2018_LU58_14	33733634	33382031	98.96%	333	15.0	SAMN15641425	PRJNA648422
2018_LU59_14	34251217	33590996	98.07%	344	15.5	SAMN15641426	PRJNA648422
2018_LU60_14	49360576	48723737	98.71%	342	21.7	SAMN15641420	PRJNA648422
2014TOR002	24883159	24460957	98.30%	358	11.1	SAMN14119528	PRJNA607000
2014TOR004	30250814	29679760	98.11%	374	13.2	SAMN14119529	PRJNA607000
2014TOR007	21809809	21341610	97.85%	347	9.6	SAMN14119530	PRJNA607000
2014TOR008	22361586	21972349	98.26%	353	10.1	SAMN14119531	PRJNA607000
2014TOR010	23359906	22986635	98.40%	337	10.6	SAMN14119532	PRJNA607000
2014TOR011	26345018	25927527	98.42%	339	11.9	SAMN14119533	PRJNA607000
2014ABO003	49325534	48626311	98.58%	376	20.8	SAMN14119523	PRJNA607000
2014COV001	37518618	36955260	98.50%	397	16.6	SAMN14119524	PRJNA607000
2014COV002	35254903	34791791	98.69%	368	15.4	SAMN14119525	PRJNA607000
2014COV004	40352819	39811383	98.66%	393	17.5	SAMN14119526	PRJNA607000
2014COV006	51113469	50262179	98.33%	395	22.1	SAMN14119527	PRJNA607000
02SELI0080	82763362	81657940	98.66%	333	26.1	SAMN14119561	PRJNA607000
02SELI0084	21808304	21493952	98.56%	202	9.0	SAMN14119562	PRJNA607000
02SELI0085	30605528	30060922	98.22%	200	11.8	SAMN14119563	PRJNA607000
04SELI0084	30251238	24498830	80.98%	281	11.0	SAMN14119571	PRJNA607000
06SELI0015	27654582	25316255	91.54%	380	11.1	SAMN08461385	PRJNA433010
06SELI0030	23557112	22800252	96.79%	383	9.9	SAMN08461386	PRJNA433010
10SELI_O124	18844890	18015096	95.60%	233	8.0	SAMN08461412	PRJNA433010
10SELI_O160	22019740	20928651	95.04%	218	8.8	SAMN08461416	PRJNA433010
12SELI0021	75285736	40975153	54.43%	252	15.8	SAMN08461425	PRJNA433010
12SELI0072	63272870	42715847	67.51%	226	16.5	SAMN08461429	PRJNA433010

Supplementary Table 3. Metadata for sample subset from The Anopheles gambiae 1000 Genomes Consortium.

ox_code	population	country	location	year	m_s	sex	n_sequences	mean_coverage	ebi_sample_acc	latitude	longitude
AA0044-C	GHcol	Ghana	Takoradi	2012	M	F	103044262	33.67	ERS311910	4.91217	-1.77397
AA0055-C	GHcol	Ghana	Takoradi	2012	M	F	95721108	30.93	ERS311903	4.91217	-1.77397
AA0056-C	GHcol	Ghana	Takoradi	2012	M	F	102828780	31.52	ERS311911	4.91217	-1.77397
AA0064-C	GHcol	Ghana	Twifo Praso	2012	M	F	100028190	31.61	ERS311880	5.60858	-1.54926
AA0067-C	GHcol	Ghana	Takoradi	2012	M	F	101513748	32.58	ERS311904	4.91217	-1.77397
AA0074-C	GHcol	Ghana	Madina	2012	M	F	104975078	33.95	ERS311960	5.66849	-0.21928
AA0075-C	GHcol	Ghana	Twifo Praso	2012	M	F	98566392	31.67	ERS311873	5.60858	-1.54926
AA0080-C	GHcol	Ghana	Takoradi	2012	M	F	102825240	31.97	ERS311913	4.91217	-1.77397
AA0098-C	GHcol	Ghana	Madina	2012	M	F	93843990	30.93	ERS311962	5.66849	-0.21928
AA0103-C	GHcol	Ghana	Takoradi	2012	M	F	102625356	33.23	ERS311907	4.91217	-1.77397
AA0109-C	GHcol	Ghana	Madina	2012	M	F	104581480	33.66	ERS311955	5.66849	-0.21928
AA0113-C	GHcol	Ghana	Twifo Praso	2012	M	F	97363646	30.68	ERS311892	5.60858	-1.54926
AA0115-C	GHcol	Ghana	Takoradi	2012	M	F	101554368	30.96	ERS311908	4.91217	-1.77397
AA0132-C	GHcol	Ghana	Madina	2012	M	F	101639112	32.39	ERS311949	5.66849	-0.21928
AA0139-C	GHcol	Ghana	Madina	2012	M	F	97694602	31.65	ERS311970	5.66849	-0.21928
AB0089-C	BFcol	Burkina Faso	Bana	2012	M	F	145350454	41.36	ERS224031	11.233	-4.472
AB0091-C	BFcol	Burkina Faso	Bana	2012	M	F	98833426	29.97	ERS224065	11.233	-4.472
AB0094-C	BFcol	Burkina Faso	Bana	2012	M	F	94712888	31.76	ERS224057	11.233	-4.472
AB0101-C	BFcol	Burkina Faso	Bana	2012	M	F	102477906	28.86	ERS223977	11.233	-4.472
AB0114-C	BFcol	Burkina Faso	Bana	2012	M	F	163544496	51.5	ERS224023	11.233	-4.472
AB0115-C	BFcol	Burkina Faso	Bana	2012	M	F	131902346	42.35	ERS224774	11.233	-4.472
AB0182-C	BFcol	Burkina Faso	Bana	2012	M	F	99125704	31.73	ERS223922	11.233	-4.472
AB0196-C	BFcol	Burkina Faso	Bana	2012	M	F	109112972	33.59	ERS224007	11.233	-4.472
AB0212-C	BFcol	Burkina Faso	Bana	2012	M	F	97935414	32.14	ERS223909	11.233	-4.472
AB0221-C	BFcol	Burkina Faso	Souroukoudinga	2012	M	F	109793482	33.19	ERS223757	11.235	-4.535
AB0237-C	BFcol	Burkina Faso	Souroukoudinga	2012	M	F	99769250	32.98	ERS224094	11.235	-4.535
AB0248-C	BFcol	Burkina Faso	Souroukoudinga	2012	M	F	131578688	41.29	ERS224785	11.235	-4.535
AB0257-C	BFcol	Burkina Faso	Pala	2012	M	F	112350788	36.13	ERS224174	11.15	-4.235
AB0276-C	BFcol	Burkina Faso	Pala	2012	M	F	99259424	31.57	ERS224319	11.15	-4.235

AB0282-C	BFcol	Burkina Faso	Pala	2012	M	F	126481642	33.21	ERS224181	11.15	-4.235
AR0001-C	AOcol	Angola	Luanda	2009	M	F	113692568	34.63	ERS224790	-8.821	13.291
AR0018-C	AOcol	Angola	Luanda	2009	M	F	39967272	12.87	ERS224234	-8.821	13.291
AR0019-C	AOcol	Angola	Luanda	2009	M	F	92841496	30.47	ERS224138	-8.821	13.291
AR0020-C	AOcol	Angola	Luanda	2009	M	F	89507990	27.64	ERS224267	-8.821	13.291
AR0036-C	AOcol	Angola	Luanda	2009	M	F	91177826	28.77	ERS224799	-8.821	13.291
AR0044-C	AOcol	Angola	Luanda	2009	M	F	91597590	28.16	ERS224797	-8.821	13.291
AR0045-C	AOcol	Angola	Luanda	2009	M	F	116771594	36.22	ERS224327	-8.821	13.291
AR0064-C	AOcol	Angola	Luanda	2009	M	F	88506992	28.54	ERS224795	-8.821	13.291
AR0069-C	AOcol	Angola	Luanda	2009	M	F	102197788	34.18	ERS224183	-8.821	13.291
AR0071-C	AOcol	Angola	Luanda	2009	M	F	96425146	29.99	ERS224194	-8.821	13.291
AR0076-C	AOcol	Angola	Luanda	2009	M	F	86444090	27.6	ERS224176	-8.821	13.291
AR0078-C	AOcol	Angola	Luanda	2009	M	F	70866952	23.56	ERS224040	-8.821	13.291
AR0088-C	AOcol	Angola	Luanda	2009	M	F	98923748	31.98	ERS224215	-8.821	13.291
AR0090-C	AOcol	Angola	Luanda	2009	M	F	119250566	39.05	ERS224106	-8.821	13.291
AR0099-C	AOcol	Angola	Luanda	2009	M	F	74509650	26.08	ERS224068	-8.821	13.291
AV0038-C	GNcol	Guinea	Koundara	2012	M	F	98739380	30.87	ERS224602	8.5	-9.417
AV0040-C	GNcol	Guinea	Koundara	2012	M	F	116667822	35.42	ERS224634	8.5	-9.417
AV0042-C	GNcol	Guinea	Koundara	2012	M	F	116397790	35.68	ERS224593	8.5	-9.417
AV0046-C	GNcol	Guinea	Koundara	2012	M	F	68296222	21.57	ERS224863	8.5	-9.417
AY0006-C	CIcol	Cote d'Ivoire	Tiassale	2012	M	F	92062494	29.87	ERS311783	5.89839	-4.82293
AY0013-C	CIcol	Cote d'Ivoire	Tiassale	2012	M	F	98003590	31.85	ERS311839	5.89839	-4.82293
AY0025-C	CIcol	Cote d'Ivoire	Tiassale	2012	M	F	95844526	29.28	ERS311848	5.89839	-4.82293
AY0029-C	CIcol	Cote d'Ivoire	Tiassale	2012	M	F	90200192	29.58	ERS311793	5.89839	-4.82293
AY0032-C	CIcol	Cote d'Ivoire	Tiassale	2012	M	F	94713402	23.61	ERS311817	5.89839	-4.82293
AY0042-C	CIcol	Cote d'Ivoire	Tiassale	2012	M	F	100457110	22.44	ERS311810	5.89839	-4.82293
AY0045-C	CIcol	Cote d'Ivoire	Tiassale	2012	M	F	97984630	30.7	ERS311834	5.89839	-4.82293
AY0053-C	CIcol	Cote d'Ivoire	Tiassale	2012	M	F	93312306	30.81	ERS311811	5.89839	-4.82293
AY0055-C	CIcol	Cote d'Ivoire	Tiassale	2012	M	F	112393152	37.35	ERS311827	5.89839	-4.82293
AY0056-C	CIcol	Cote d'Ivoire	Tiassale	2012	M	F	105331900	34.18	ERS311835	5.89839	-4.82293
AY0062-C	CIcol	Cote d'Ivoire	Tiassale	2012	M	F	102271052	31.96	ERS311796	5.89839	-4.82293
AY0065-C	CIcol	Cote d'Ivoire	Tiassale	2012	M	F	99574902	30.24	ERS311820	5.89839	-4.82293

AY0068-C	CIcol	Cote d'Ivoire	Tiassale	2012	M	F	95617762	30.58	ERS311844	5.89839	-4.82293
AY0076-C	CIcol	Cote d'Ivoire	Tiassale	2012	M	F	89669580	27.58	ERS311821	5.89839	-4.82293
AY0090-C	CIcol	Cote d'Ivoire	Tiassale	2012	M	F	96210618	30.94	ERS311846	5.89839	-4.82293
