# Supplementary material for: The origin of island populations of the African malaria mosquito, Anopheles coluzzii
Source: Commun Biol. 2021 May 26;4:630. doi: 10.1038/s42003-021-02168-0 (PMC8155153; doi:10.1038/s42003-021-02168-0)
Supplement: Supplementary file 2 — Reporting Summary [file 42003_2021_2168_MOESM2_ESM.pdf]

nature research

Corresponding author(s): Gregory Lanzaro

Last updated by author(s): Apr 9, 2021

Reporting Summary

Nature Research wishes to improve the reproducibility of the work that we publish. This form provides structure for consistency and transparency in reporting. For further information on Nature Research policies, see our Editorial Policies and the Editorial Policy Checklist.

Statistics

For all statistical analyses, confirm that the following items are present in the figure legend, table legend, main text, or Methods section.

n/a	Confirmed	
☐	☒	The exact sample size (*n*) for each experimental group/condition, given as a discrete number and unit of measurement
☐	☒	A statement on whether measurements were taken from distinct samples or whether the same sample was measured repeatedly
☐	☒	The statistical test(s) used AND whether they are one- or two-sided<br>*Only common tests should be described solely by name; describe more complex techniques in the Methods section.*
☐	☒	A description of all covariates tested
☐	☒	A description of any assumptions or corrections, such as tests of normality and adjustment for multiple comparisons
☐	☒	A full description of the statistical parameters including central tendency (e.g. means) or other basic estimates (e.g. regression coefficient) AND variation (e.g. standard deviation) or associated estimates of uncertainty (e.g. confidence intervals)
☐	☒	For null hypothesis testing, the test statistic (e.g. *F*, *t*, *r*) with confidence intervals, effect sizes, degrees of freedom and *P* value noted<br>*Give P values as exact values whenever suitable.*
☐	☒	For Bayesian analysis, information on the choice of priors and Markov chain Monte Carlo settings
☐	☒	For hierarchical and complex designs, identification of the appropriate level for tests and full reporting of outcomes
☐	☒	Estimates of effect sizes (e.g. Cohen's *d*, Pearson's *r*), indicating how they were calculated

Our web collection on statistics for biologists contains articles on many of the points above.

Software and code

Policy information about availability of computer code

Data collection	Not applied
Data analysis	Trimmomatic v0.36, BWA-MEM v0.7.15, Sambamba, Freebayes v1.2.0 , vcffilter v1.0.0rc2, ape 5.4, BCFtools v1.9, scikit-allel v1.2.0, ADMIXTURE v1.3.0, CLUMPAK, VCFtools v1.16, PLINK v1.9, MSMC2 v2.0.2, SHAPEIT2 v2.9

For manuscripts utilizing custom algorithms or software that are central to the research but not yet described in published literature, software must be made available to editors and reviewers. We strongly encourage code deposition in a community repository (e.g. GitHub). See the Nature Research guidelines for submitting code & software for further information.

Data

Policy information about availability of data

All manuscripts must include a data availability statement. This statement should provide the following information, where applicable:

- Accession codes, unique identifiers, or web links for publicly available datasets
- A list of figures that have associated raw data
- A description of any restrictions on data availability

New whole genome sequence data included in this study are deposited in NCBI GenBank with accession numbers SAMN17251765, SAMN15641374-SAMN15641426 under BioProject ID PRJNA648422.

Field-specific reporting

Please select the one below that is the best fit for your research. If you are not sure, read the appropriate sections before making your selection.

[x] Life sciences [ ] Behavioural & social sciences [ ] Ecological, evolutionary & environmental sciences

For a reference copy of the document with all sections, see nature.com/documents/nr-reporting-summary-flat.pdf

Life sciences study design

All studies must disclose on these points even when the disclosure is negative.

Sample size	Sample size was determine to achieve a wide geographic coverage of Anopheles coluzzii.
Data exclusions	No data were excluded.
Replication	No unusual attempts.
Randomization	Not relevant, because the affiliation with the geographical groups was the essence of the study.
Blinding	Blinding was not relevant for this study.

Reporting for specific materials, systems and methods

We require information from authors about some types of materials, experimental systems and methods used in many studies. Here, indicate whether each material, system or method listed is relevant to your study. If you are not sure if a list item applies to your research, read the appropriate section before selecting a response.

Materials & experimental systems

n/a	Involved in the study
[x]	[ ] Antibodies
[x]	[ ] Eukaryotic cell lines
[x]	[ ] Palaeontology and archaeology
[ ]	[x] Animals and other organisms
[x]	[ ] Human research participants
[x]	[ ] Clinical data
[x]	[ ] Dual use research of concern

Methods

n/a	Involved in the study
[x]	[ ] ChIP-seq
[x]	[ ] Flow cytometry
[x]	[ ] MRI-based neuroimaging

Animals and other organisms

Policy information about studies involving animals; ARRIVE guidelines recommended for reporting animal research

Laboratory animals	Mosquitoes from the species Anopheles coluzzii were used.
Wild animals	No animals were brought into captivity.
Field-collected samples	Mosquitoes collected from the field were sacrificed on site.
Ethics oversight	No ethical approval was required.

Note that full information on the approval of the study protocol must also be provided in the manuscript.
